# The roles of emotional intelligence, neuroticism, and academic stress on the relationship between psychological distress and burnout in medical students

**DOI:** 10.1186/s12909-021-02733-5

**Published:** 2021-05-22

**Authors:** Muhamad Saiful Bahri Yusoff, Siti Nurma Hanim Hadie, Mohd Azhar Mohd Yasin

**Affiliations:** 1 Department of Medical Education, School of Medical Sciences, Universiti Sains Malaysia, Kota Bharu, Kelantan Malaysia; 2 Department of Anatomy, School of Medical Sciences, Universiti Sains Malaysia, Kota Bharu, Kelantan Malaysia; 3 Deparment of Psychiatry, School of Medical Sciences, Universiti Sains Malaysia, Kota Bharu, Kelantan Malaysia

**Keywords:** Burnout, Psychological distress, Emotional intelligence, Neuroticism, Academic stress

## Abstract

**Background:**

Stress and burnout commonly threaten the mental health of medical students in Malaysia and elsewhere. This study aimed to explore the interrelations of psychological distress, emotional intelligence, personality traits, academic stress, and burnout among medical students.

**Methods:**

A cross-sectional study was conducted with 241 medical students. Validated questionnaires were administered to measure burnout, psychological distress, emotional intelligence, personality traits, and academic stress, respectively. A structural equation modelling analysis was performed by AMOS.

**Results:**

The results suggested a structural model with good fit indices, in which psychological distress and academic stress were noted to have direct and indirect effects on burnout. The burnout levels significantly increased with the rise of psychological distress and academic stress. Neuroticism was only found to have significant indirect effects on burnout, whereby burnout increased when neuroticism increased. Emotional intelligence had a significant direct effect on lowering burnout with the incremental increase of emotional intelligence, but it was significantly reduced by psychological distress and neuroticism.

**Conclusion:**

This study showed significant effects that psychological distress, emotional intelligence, academic stress, and neuroticism have on burnout. Academic stress and neuroticism significantly increased psychological distress, leading to an increased burnout level, while emotional intelligence had a significant direct effect on reducing burnout; however, this relationship was compromised by psychological distress and neuroticism, leading to increased burnout. Several practical recommendations for medical educators, medical students, and medical schools are discussed.

## Introduction

Psychological distress is an unpleasant emotional state experienced by individuals in response to demands that cause mental disturbances [1]. Psychological distress is a multi-facet construct that correlates with poor mental health and function [2, 3]. Literature has shown psychological distress is more prevalent in medical students than in the public [4, 5]. However, before the medical training start, medical students have shown a similar level of psychological health as compared to the public [5–8]. In comparison to undergraduate students across 15 courses, the students enrolled in medicine and health science courses showed the highest psychological distress scores [9]. These facts suggest medical training is challenging and demanding for young medical students [10]. Given its prevalence and the serious nature of the consequences, understanding the underlying factors contributing to psychological distress is imperative for necessary intervention.

Mental health implies a state of wellbeing enabling individuals to realise their abilities, cope with the normal stresses of life, work productively, and contribute to their communities [11]. Unfortunately, some components of training have unintended and detrimental impacts on students’ mental health. A high proportion of students experience high academic-related stress [12–15]. The negative factors contributing to psychological distress among medical students are related to academic stress, such as course activities, course load, examinations, teaching and learning hassles, placement hassles, and high workload [14, 16]. Empirical evidence demonstrates that psychological distress was positively correlated with burnout [3], which certainly affects performance and mental health [17]. Burnout can be conceptualised as a syndrome of emotional exhaustion, depersonalization, and a low sense of personal accomplishment [18] that eventually leads to detrimental consequences to mental health and psychological function [17, 19]. The prevalence of burnout among medical students during medical training is high at approximately 43.3% [19], in which 35–45% of medical students had high emotional exhaustion, 26–38% experienced high depersonalization, and 45–56% had symptoms suggestive of burnout [17]. These facts demonstrate academic stress contributes to psychological distress and burnout, which may contribute to personal and professional consequences, for instance, increased rate of medical error, malpractice suits, and increased likelihood of physician suicide [19–21].

Literature shows that medical students with a specific personality, especially neuroticism traits, were more vulnerable to developing psychological distress and burnout [19]. Neuroticism is generally characterised by a tendency to experience negative feelings and is linked with emotional instability, distress, moodiness, irritability, poor coping ability, and sadness [22, 23]. Likewise, in the medical context, personality traits are associated with several important areas, which include the approach to work, mental health, career success, learning approach, and academic performance of medical students and professionals [24, 25]. A longitudinal study reported that neuroticism was the strongest factor of psychological distress for medical students during stressful periods, such as the final examination [26]. Besides, a study had also shown that burnout was strongly linked to neuroticism and it explained more variance in burnout than work stress [27]. These facts suggest individuals with high neuroticism are more vulnerable to developing psychological distress and burnout. The significance of personality traits may have been underestimated in burnout research among medical students, hence this study was an attempt to close this research gap.

Moreover, there is considerable evidence showing that emotional intelligence (EI) is a determinant of success in a variety of occupational settings [28–30]. EI refers to the ability to perceive, express, understand, motivate, control, and regulate emotion [30–33]. A recent systematic review on EI in medicine revealed that a higher EI positively contributed to important outcomes in the competence of future doctors [29], and EI prior to medical training predicted psychological health and academic performance during medical training [26]. For example, a cross-sectional study found emotional intelligence negatively correlated with depression and anxiety levels [34], and a longitudinal study found that self-appraisal emotion was the only EI dimension related to burnout one year later [35]. These facts suggest that EI plays a significant role in several areas that are related to the psychological health of future doctors.

Considering all facts related to the effects that personality, EI, and academic life might have on psychological distress and burnout, this study attempted to provide a best fit structural model for the interrelations of burnout with psychological distress, academic stress, personality, and EI. This study hypothesized that (a) psychological distress is a predisposing or contributing factor to burnout; (b) academic and psychological stress on the same side or in the same role lead to burnout; and (c) neuroticism and emotional intelligence are predictors or mediators of the link between psychological distress and burnout.

## Method

A cross-sectional study was conducted, and a purposive sampling method was applied with 300 second-year medical students in the academic session of 2016 and 2017. The acceptable sample size for structural equation modelling is 200 to 300 samples [36]. The data collection was carried out immediately after the final preclinical examination. Informed consent was obtained, and participation in this study was voluntary that would not affect participants’ academic progression.

The medical students studied a five-year medical program based on the SPICES (i.e., student-oriented, problem-based, integrated, community-oriented, electives, self-learning, and systematic learning) curriculum model. The medical program was organized into the pre-clinical phase (first and second year) and the clinical phase (third, fourth, and fifth year). Medical students in the pre-clinical phase learn the basic and applied knowledge related to the normal human being and early clinical exposure to common pathological conditions. Medical students in the clinical phase learn clinical sciences and skills in a workplace setting.

Burnout was measured by the Copenhagen Burnout Inventory (CBI) consists of 19 items. It is the newest and public domain tool developed to assess the core features of burnout (fatigue and exhaustion) concerning personal life (personal burnout), work (work-related burnout), and service to clients (client-related burnout) [37–39]. The CBI was validated in the medical student population [40], which has three domains; personal, work-related, and client-related burnout [38, 40]. The internal structure reliability of the CBI was good with Cronbach’s alpha ranging from 0.83 to 0.87 [40]. Reversed scoring was applied in positively worded items, and high scores indicated high levels of burnout.

Psychological distress was measured using the 21-item Depression Anxiety Stress Scale (DASS-21). Researchers have used the DASS-21 to measure symptoms of depression, anxiety, and stress as well as overall psychological distress, in which a high score indicates poor psychological health [41–45]. Its validity and reliability among student samples have been well established in previous studies [42, 44–46]. The internal consistency coefficients of depression, anxiety, and stress scales ranged between 0.81 and 0.97 [46].

EI was measured using the 17-item USM Emotional Quotient Inventory (USMEQ-17), which is a valid and reliable tool for measuring EI in medical student samples as it demonstrated high internal consistency (Cronbach’s alpha was greater than 0.7) and good construct validity [47–50]. The assessment consists of personal competence and social competence, both domains represented by global EI are together the ability to perceive, express, understand, motivate, control, and regulate emotion.

The 15-item USM Personality Traits Inventory (USMaP-15) measures the five-factor personality traits, which are openness, conscientiousness, extroversion, agreeableness, and neuroticism [51–53]. It is a valid and reliable tool to measure personality traits in medical student samples as it demonstrated a stable internal consistency (Cronbach’s alpha) that ranged from 0.63–0.83 and a good construct [51, 52, 54], indicating an acceptable to high level of internal consistency and consistency across time intervals and occasions.

The 20-item Medical Student Stressors Questionnaire (MSSQ-20) measures academic stress [55, 56]. The MSSQ-20 has six domains, including academic, interpersonal, teaching and learning, social, drive/desire, and group activity. It is a self-reported self-scoring instrument that requires medical students to rate the intensity of stress caused by each source. The internal consistency (Cronbach’s alpha) for the MSSQ-20 was more than 0.8 and ranged from 0.55 to 0.97 for each MSSQ construct [56]. The MSSQ-20 has stable internal consistency over multiple measurements across different time intervals [57].

**Fig. 1:**
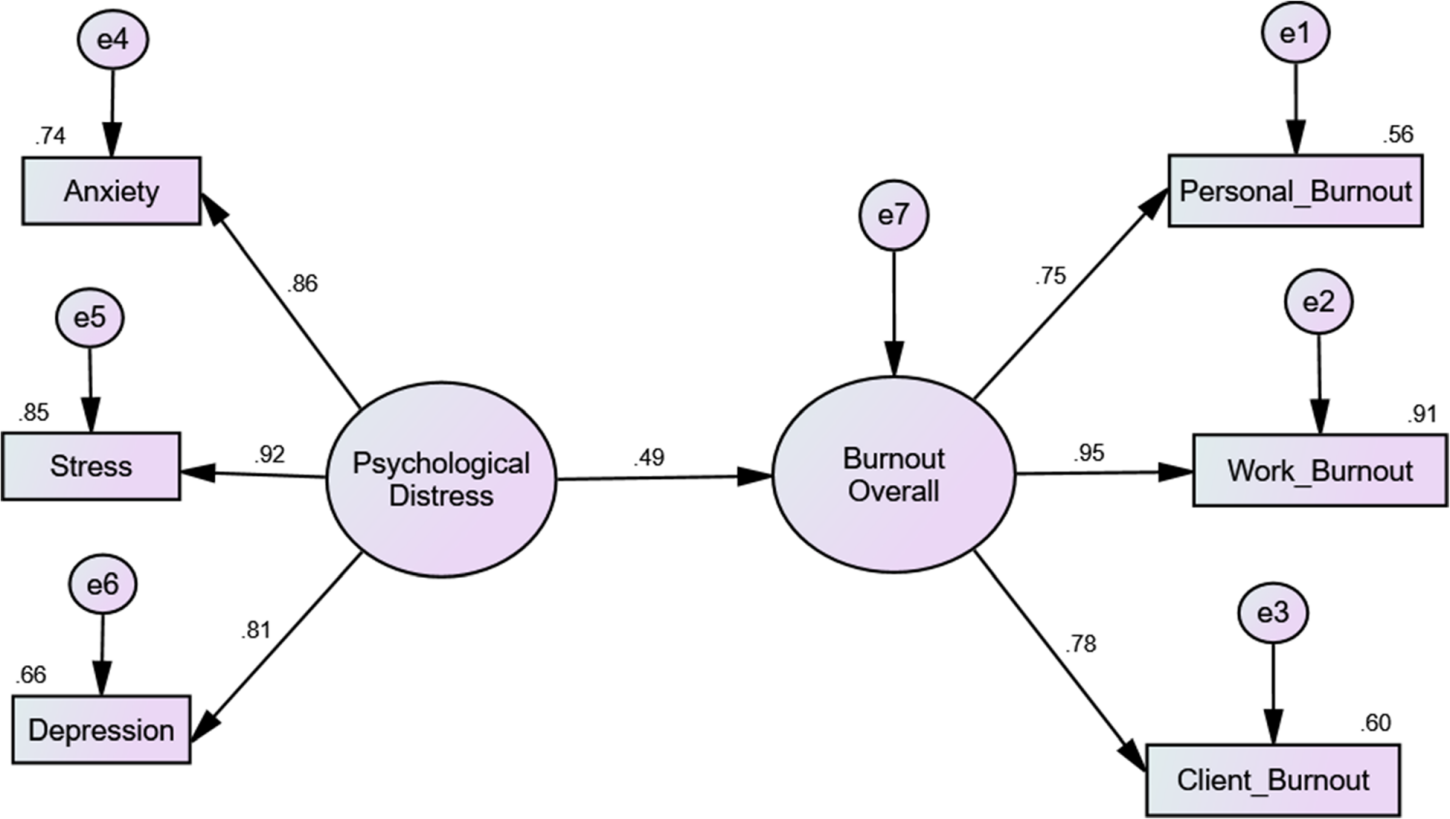
Structural equation modelling (standardised estimates) of the psychological distress-burnout relationship. *(e = error; the decimal value estimates contribution of an item to the construct’s variance)*

A descriptive analysis of the demographic data was performed using the Statistical Package for Social Sciences (SPSS) version 20. Structural equation modelling (SEM) was performed on the samples with complete responses to all five inventories to examine the interrelations between observable variables in the proposed model (Fig. 1). CFA and SEM were performed using the Analysis of Moment Structure (AMOS) software. The latent constructs and the proposed model were considered fit if all the goodness of fit indices achieved the minimal requirement [36], as stated in Table 1.

**Table 1:** The goodness of fit indices used to signify model fitness

Name of category	Name of index	Level of acceptance
Absolute fit^a^	Root mean square of error approximation (RMSEA)	less than 0.08
	Goodness of fit index (GFI)	more than 0.9
Incremental fit^b^	Comparative fit index (CFI)	more than 0.9
	Tucker-Lewis index (TLI)	more than 0.9
	Normed fit index (NFI)	more than 0.9
Parsimonious fit^c^	Chi-square/degree of freedom (Chisq/df)	less than 5

*Note.*^a^Absolute fit: Measures overall goodness of fit for both the structural and measurement models collectively. This type of measure does not make any comparison to a specified null model (incremental fit measure) or adjust for the number of parameters in the estimated model (parsimonious fit measure). ^b^Incremental fit: Measures goodness of fit that compares the current model to a specified “null” (independence) model to determine the degree of improvement over the null model. ^c^Parsimonious fit: Measures goodness of fit representing the degree of model fit per estimated coefficient. This measure attempts to correct for any “overfitting” of the model and evaluates the parsimony of the model compared to the goodness of fit

## Results

Out of 300, 241 second-year medical students [*n* (%)_2016 batch_ = 141 (58.5%); *n* (%)_2017 batch_ = 100 (41.5%)] responded completely to the five inventories. The majority of respondents were 63.5% female (*n* = 153) and 49.4% non-Malay (*n* = 122), and the mean age was about 21 years (*M* = 21.8).

The goodness of fit indices for the psychological distress-burnout relationship and the mediating effects of academic stress, neuroticism, and emotional intelligence are summarised in Table 2. The direct, indirect, and total effects of the model paths are shown in Tables 3 and 4.

**Table 2:** The goodness of fit indices for supporting the best fit model

Model	*χ*^2^ statistics (*df*)	*p*-value	The goodness of fit indices					
			*χ*^2^/*df*	RMSEA	GFI	CFI	NFI	TLI
1. Model 1 (Fig. 1)	17.48 (8)	0.025	2.186	0.070	0.976	0.989	0.980	0.980
2. Model 2 (Fig. 2)	62.78 (29)	< 0.001	2.165	0.070	0.951	0.973	0.952	0.958

*Note. χ* ^2^/*df* Chi-square/degree of freedom, *RMSEA* root mean square of error approximation, *GFI* goodness of fit index, *CFI* comparative fit index, *NFI* normed fit index, *TLI* Tucker–Lewis index

**Table 3:** The estimates of standardised and unstandardised regression weights of academic stress, neuroticism, and emotional intelligence on the psychological distress-burnout relationship

Independent variables	Dependent variables	β	B	SE	*p*-values
Psychological distress	Burnout	0.344	0.160	0.037	< 0.001
Emotional intelligence		−0.176	−1.376	0.483	0.004
Academic stress		0.168	0.781	0.309	0.012
Academic stress	Psychological distress	0.384	3.846	0.584	< 0.001
Neuroticism		0.390	1.324	0.198	< 0.001
Neuroticism	Academic stress	0.202	0.068	0.021	0.001
Psychological distress	Emotional intelligence	−0.241	−0.014	0.004	< 0.001
Neuroticism		−0.394	−0.079	0.016	< 0.001

β = standardised regression weights; B unstandardised regression weights; SE = standard error

**Table 4:** The unstandardised and standardised estimates of direct, indirect, and total effects of academic stress, neuroticism, and emotional intelligence on the psychological distress-burnout relationship

Parameter	Independent variable	Dependent variable	Total (L, U)	Direct (L, U)	Indirect (L, U)
Unstandardised	Psychological distress	Burnout	0.179 (0.099, 0.276)**	0.160 (0.073, 0.258)**	0.020 (0.003, 0.055)*
	Academic stress		1.470 (0.838, 2.069)**	0.781 (0.027, 1.424)*	0.689 (0.373, 1.114)**
	Emotional intelligence		−1.376 (−2.854, −0.091)*	−1.376 (− 2.854, − 0.091)*	–
	Neuroticism		0.447 (0.261, 0.652)**	–	0.447 (0.261, 0.652)**
Standardised	Psychological distress	Burnout	0.386 (0.214, 0.557)**	0.344 (0.141, 0.525)**	0.042 (0.009, 0.113)*
	Academic stress		0.316 (0.173, 0.429)**	0.168 (0.006, 0.300)*	0.148 (0.085, 0.231)**
	Emotional intelligence		−0.176 (−0.376, −0.014)*	−0.176 (− 0.376, − 0.014)*	–
	Neuroticism		0.283 (0.184, 0.378)**	–	0.283 (0.184, 0.378)**

*Note.* Bootstrap (1000) with 95% bias-corrected confidence interval: L = lower bound; U = upper bound;

**p-value < 0.01; *p-value < 0.05

### Psychological distress is a predisposing and contributing factor to burnout

The psychological distress-burnout relationship achieved model fit (Fig. 1, Table 2: Model 1). The relationship between psychological distress and burnout was significant (β = 0.494, B = 0.228, SE = 0.035, *p*-value < 0.001). When psychological distress went up by 1 unit or standard deviation (SD), burnout went up by 0.228 units or 0.494 SDs. Of importance, psychological distress significantly contributed to the burnout level of medical students.

### Academic and psychological stress on the same side and in the same role lead to burnout

**Fig. 2:**
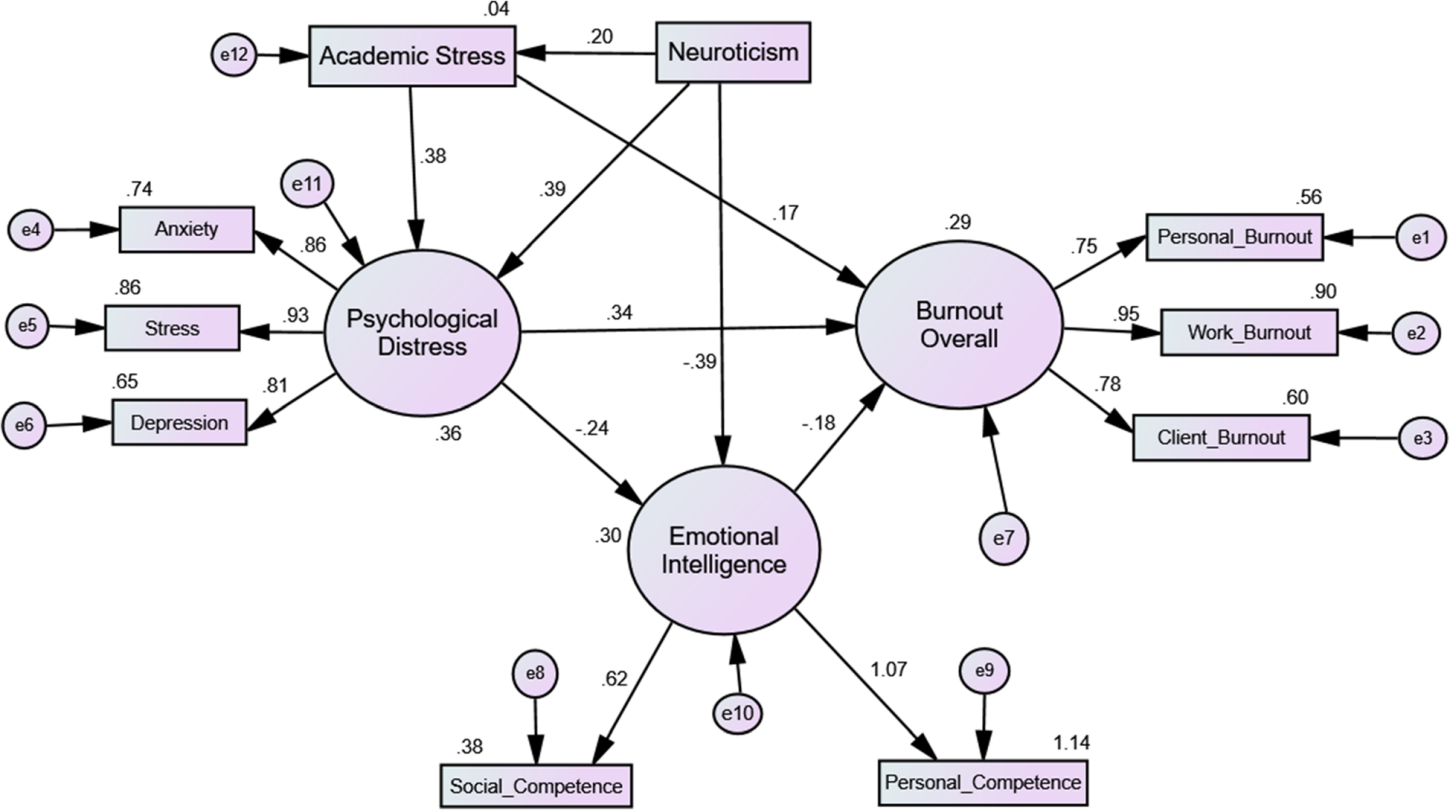
Structural equation modelling (standardised estimates) for the mediating effects of academic stress, neuroticism, and emotional intelligence on the psychological distress-burnout relationship. *(e = error; the decimal value estimates contribution of an item to the construct’s variance)*

The psychological distress significantly increased burnout levels and decreased EI (Table 3, Fig. 2) When psychological distress increased by 1 unit or SD, burnout increased by 0.160 units or 0.344 SDs. Significantly, the effects of psychological distress on burnout were reduced after including EI, academic stress, and neuroticism into the SEM, as compared to Model 1 (Fig. 1). EI significantly reduced the burnout level, when EI increased, burnout decreased. Furthermore, academic stress significantly increased psychological distress and burnout levels. Thus, when academic stress increased, psychological distress and burnout increased. Additionally, neuroticism significantly increased academic stress and psychological distress and decreased EI. When neuroticism increased, academic stress and psychological distress also increased.

### Neuroticism and emotional intelligence are predictors or mediators of the link between psychological distress and burnout

The results showed a significant direct effect of psychological distress on burnout (Table 4). However, the effect of psychological distress on burnout was significantly mediated by EI (Fig. 2). Additionally, psychological distress significantly decreased EI, but EI directly decreased burnout levels (Tables 3 and 4. In other words, emotional intelligence was a protective factor for burnout.

Additionally, a significant effect was shown with academic stress and neuroticism in the relationship between psychological distress and burnout (Fig. 2; Tables 3 and 4). Both academic stress and neuroticism increased psychological distress, thereby contributing to the increased burnout level. Academic stress showed both direct and indirect effects on burnout; although, neuroticism only showed an indirect effect on burnout. In other words, academic stress was a predictor of the relationship between psychological distress and burnout; however, neuroticism was a predisposing contributing factor to burnout.

The results also identified that EI was significantly reduced by psychological distress and neuroticism, indicating both were negative predictors of EI, which suggests that psychological distress and neuroticism increased burnout levels by reducing EI levels. These results indicated the significant effects and paths that psychological distress, emotional intelligence, academic stress, and neuroticism have on burnout. Academic stress and neuroticism significantly increase psychological distress, leading to the escalation of burnout levels, while EI has a significant effect on reducing burnout; however, this is negatively affected by psychological distress and neuroticism, leading to increased burnout.

## Discussion

This study contributes several important findings to the current body of knowledge. First, psychological distress predominantly has a direct effect on escalating the burnout level of medical students. Second, academic stress has direct and indirect effects (mainly mediated through psychological distress) on increasing the burnout level of medical students during stressful events. Third, neuroticism predominantly has an indirect effect, mediated through psychological distress and academic stress, on increasing medical students’ burnout levels during stressful periods. Fourth, EI mainly demonstrates a direct effect on reducing the burnout level of medical students during stressful events. Lastly, EI was significantly compromised by the increase of psychological distress and neuroticism, leading to burnout.

First, psychological distress predominantly had direct effects on escalating the burnout level of medical students. It had the greatest direct standardised effect in positively predicting burnout – making it the strongest predictor of burnout in medical students. This finding corresponds to previous studies that showed a significant positive correlation between psychological distress and burnout [3], a significant negative correlation between psychological wellbeing and burnout [58], a significant positive correlation between burnout and depression [59–63], a significant correlation between anxiety and burnout [63–65], and the strong association of burnout severity with the prevalence of depression [66]. These indicate that psychological distress is the major predictor of burnout, highlighting the importance of reducing unnecessary psychological pressures (sources of stress that are not needed to be introduced), thus leading to better psychological health and eventually reducing burnout in medical students [67, 68]. Designing a systematic support system, for instance, a peer-support system, to support medical students experiencing difficulty may improve their psychological distress by helping to reduce unnecessary psychological pressures [12, 19].

Second, academic stress demonstrated direct and indirect effects (mainly mediated through psychological distress) on increasing the burnout level of medical students during stressful events. Academic stress had the second greatest total effect on burnout. This finding is consistent with previous studies that reported daily hassles positively correlated with burnout [63], academic stress negatively correlated with personal wellness [69], perceived stress associated with burnout [70], and academic stress as the most predictive of burnout [71]. These facts suggest that psychological wellbeing is negatively affected by high academic stress due to the demands of medical training [69, 72]. Taib et al. (2020) explained that “Most budding doctors believe that hard work, sweat and dedication would lead to successful careers” (p.66). Unfortunately, many trainees experience medical and mental health problems, which have become more apparent and overwhelming following the demands of clinical training. Thus, empathetic and healing relationships are vital rather than suspicion and hatred when dealing with the unwell student [12]. It is possible that lowering superfluous academic stress by reducing unnecessary syllabus, course load, workload, and psychological pressures while fostering psychological support, a healthy learning environment, sufficient learning time, and adequate breaks would improve their psychological well-being and prevent them from developing burnout [19].

Third, neuroticism predominantly had an indirect effect (mediated through psychological distress and academic stress) on increasing medical students’ burnout levels during stressful periods. Previous studies reported that certain personality traits can contribute to stress among medical students and reduce their wellbeing [72], for instance, neuroticism demonstrated a positive correlation with emotional exhaustion and cynicism and a negative correlation with professional efficacy [73], burnout risk was strongly associated with neuroticism [74], and neuroticism positively correlated with psychological distress [19]. These findings recognize neuroticism as a negative predictor of psychological wellbeing. One possible reason is potentially due to the tendency of individuals with high neuroticism to experience negative feelings and to have the poor coping ability in stressful circumstances [75, 76], making them less suitable for medical training that is complicated and stressful [77, 78]. On that basis, medical schools should consider including neuroticism as a criterion in the recruitment of candidates into medical programs because it will influence the quality of medical graduates [79].

Fourth, EI demonstrated a direct effect on reducing the burnout level of medical students during stressful events. This finding is aligned with several studies reporting that EI scores correlate inversely with emotional exhaustion and depersonalization [80], that EI was strongly predictive of emotional exhaustion and depersonalization [80], and that higher EI scores were significantly correlated with lower burnout [81]. These facts indicate that individuals with higher EI will have a better psychological state and be less vulnerable to developing burnout. Emotionally intelligent persons know how to handle their own and others’ emotions and being able to deal with emotions effectively makes them less vulnerable to developing burnout. Hence, developing a special program to cultivate medical students’ EI could help students to face the demands and challenges of medical training, thus preventing them from developing burnout. Medical schools could also possibly include EI as a criterion for the recruitment of candidates into medical training, thus will minimise the vulnerability of students to develop burnout.

Lastly, EI was significantly compromised by the increase of psychological distress and neuroticism, which led to burnout. It was evident in the literature that EI correlated positively with psychological wellbeing and inversely with depression [80], that self-perceived stress was lower in those with higher EI [82], that psychological distress showed a negative correlation with EI scores [83], and that EI demonstrated negative correlations between anxiety, stress, and depression [84]. One important fact from these findings is the indirect mechanism through which psychological distress causes burnout is by lowering the EI of medical students. Similarly, personality contributed significantly to EI, especially neuroticism, which demonstrated the largest independent negative contribution to the increase of burnout [85] via the same indirect mechanism as psychological distress. This is a significant fact for consideration given that medical students usually have high EI but are still vulnerable to burnout if they are consistently exposed to chronic excessive psychological pressure. This is known as a wear and tear phenomenon due to the depletion of the emotional reservoir in handling chronic exposure to prolonged excessive psychological pressures [86], especially in those with high neuroticism.

Based on the SEM, several practical applications can be recommended to medical educators, students, and medical schools. First, medical educators should try their best to avoid introducing psychological pressures that are not needed to students, especially academic-related stress. For an example, medical educators should convey clear expectations on the academic requirement to students especially the assessment matters as it is the most stressful event for medical students [19]. This approach will minimise the sources of psychological distress and burnout, hence, lead to better mental health. Second, medical students should do their best to develop a positive and healthy mindset towards academic matters that will help them to thrive under pressure. One of techniques that was reported to promote a healthy and positive mindset of medical students towards sources of stress was the DEAL-based practice [19]. The DEAL-based practice is a psychoeducational tool that can help medical students to systematically and effectively manage sources of stress, thus lead to the reduction of psychological pressures [68]. Third, medical schools should introduce programs that help medical students manage their stress and develop their EI such as the DEAL-based practice and mindfulness-based stress reduction technique as these wellbeing strategies foster resilience and prevent burnout [19, 68]. Besides, having a regular assessment of these constructs (burnout, distress, academic stress, etc.) would be useful for medical schools and students as it could help educators identify medical students at risk and increase the awareness that encourages them to engage in self-care practices to avoid the need for acute intervention. Lastly, medical schools maybe should consider regularly assessing neuroticism, EI and other constructs upon entry and throughout medical training due to certain personality traits (like neuroticism) are relatively stable in middle and older adulthood; however, medical students are typically younger and still developing. Concerning scores in these areas could be seen not as a disqualifying factor but as a point of prevention or intervention. It is worth highlighting that this study showed candidates with low neuroticism and high EI will be able to handle medical training pressures in a better way, hence minimising the probability of them developing psychological distress and burnout.

It is worthy to mention this research was conducted at a medical school; therefore, any attempt to generalise the results to other settings should be done cautiously. A multi-centre and longitudinal research should be conducted in the future to validate the proposed model as distress, burnout, academic stress, EI, and even neuroticism may different at different educational settings and fluctuate over time. Besides, the sampling technique used was not the ideal method due to the limitation of the non-probability technique due to sampling bias, which may cause imprecision of the obtained results. Hence, future research should use the probability sampling technique to overcome this limitation. Lastly, it is recommended to perform subanalysis by gender and academic years in the future study to examine this model because EI may be different according to the gender, and also, if the subjects were in a different academic year, they could have a different level of distress and burnout. Despite these limitations, this research has several strengths. First, the research variables were measured by validated research tools, and the obtained results supported the measurement model fit. Second, the sample size was satisfactory for SEM; thus, the obtained results are trustworthy for the proposed structural model. Third, the analysis was conducted by standard and recommended statistical software; therefore, the obtained results can be trusted and compared with previous studies. Lastly, as far as the author is aware, this is the first attempt to describe the causal-effect relationships of burnout, psychological distress, academic stress, neuroticism, and EI through SEM.

## Conclusion

The results revealed significant effects and paths that psychological distress, EI, academic stress, and neuroticism have on burnout. Academic stress and neuroticism significantly increased psychological distress, leading to increased burnout levels, while EI had a significant direct effect on reducing burnout; however, this was compromised by psychological distress and neuroticism leading to increased burnout. This study explained the causal-effect relationships of burnout, psychological distress, academic stress, neuroticism, and EI through SEM.

## Data Availability

The datasets used and/or analysed during the current study are available from the corresponding author on reasonable request.
